# The *Fundamentals of Health Research* training for community health workers: a mixed-methods study

**DOI:** 10.3389/fpubh.2025.1610413

**Published:** 2025-06-18

**Authors:** Nicole Wolfe, Andrea Diaz, Brian Do-Golden, Esther Karpilow, Mayra Rubio-Diaz, Michele D. Kipke

**Affiliations:** ^1^Southern California Clinical and Translational Science Institute, University of Southern California, Los Angeles, CA, United States; ^2^Office of the Senior Vice President of Health Affairs, University of Southern California, Los Angeles, CA, United States; ^3^Department of Pediatrics Los Angeles, Children's Hospital of Los Angeles, Los Angeles, CA, United States

**Keywords:** community engagement, research participation, community health workers, capacity building, public health

## Abstract

**Introduction:**

Community Health Workers/Promotores de Salud (CHW/Ps) are trusted messengers embedded in their communities who bridge gaps in access to care, address misinformation, and promote health through culturally tailored education and outreach. The *Fundamentals of Health Research* training program was launched to address the gap in research engagement, and to support CHW/Ps’ professional development. This innovative initiative equips CHW/Ps with foundational knowledge about clinical research and the skills to apply that knowledge by engaging their communities in meaningful discussions about research and the importance of participation in research.

**Methods:**

This 12-h, five-part training was developed collaboratively and delivered by experienced CHW/Ps. The curriculum included interactive components such as role-playing, IRB simulations, and the design of community-relevant research projects. The program was evaluated using a mixed-methods approach, including pre- and post-training surveys, satisfaction surveys, and 3- and 12-month follow-ups.

**Results:**

Between March and September 2023, 128 CHW/Ps completed the training; 103 (80.5%) completed the 12-month follow-up. Most participants identified as Latino/Hispanic (93%) and female (96%). Quantitative findings assessed knowledge gain (46.8% increase), attitudes toward research (49.1% reported an increased likelihood of research participation post-training), self-efficacy in research communication (overall average of 8 out of 10), and application of training in community settings (53.9% reported referring people directly to research studies). Qualitative findings highlighted increased understanding of research ethics, community impact, the importance of participation in research, and persistent barriers to participation such as mistrust and misinformation.

**Conclusion:**

The *Fundamentals of Health Research* training program is an innovative, scalable community engaged model for bridging the gap between research institutions and communities. Sustained support for community-driven, evidence-based training is essential to building capacity in the CHW/P workforce, which can help to advance research access, increase participation across populations, and improve health.

## 1 Introduction

Community engagement is foundational to the success of effective public health programs by fostering trust, collaboration, and shared decision-making between communities and health institutions (1). Interventions that center community engagement have been shown to improve health behaviors, outcomes, and participants’ sense of self-efficacy (2). By actively engaging community members, public health initiatives can be tailored to meet their specific needs, ensuring greater accessibility and cultural relevance. Research shows that community engagement enhances health literacy, empowers individuals to participate in research and improves the overall effectiveness of interventions (2–4).

Community Health Workers/Promotores de Salud (CHW/Ps) play a pivotal role in this process. As trusted messengers embedded in their communities, they bridge gaps in access to care, address misinformation, and promote health through culturally tailored education and outreach (5). Their embeddedness within their communities makes them particularly effective in addressing mistrust and communicating the value of research participation in a culturally competent and accessible way. Programs that integrate CHW/Ps not only improve individual health outcomes but also help ensure that public health initiatives are accessible, culturally relevant, and responsive to the needs of those they aim to serve (3, 4).

One critical area where community engagement can have a measurable impact is clinical research. Participation by a broad range of demographics in research is essential to ensure that findings are generalizable and that medical interventions are safe and effective across different populations (6, 7). However, longstanding mistrust, rooted in unethical research and medical practices coupled with ongoing differences in healthcare access has remained the driver of the persistently low rates of participation of many communities in research (8, 9). Evidence shows that representation among research staff and culturally competent engagement strategies lead to better recruitment and retention among groups who have historically low participation rates (7).

CHW/Ps are uniquely positioned to address these challenges. As trusted community members with extensive networks who share the cultural and linguistic backgrounds of the populations they serve, they are frequently sought out for health-related guidance and information. Their strong ties to the community enable them to address a broad range of health issues providing holistic, comprehensive support (5, 10, 11). Despite their impact, CHW/Ps face structural barriers such as low wages, limited professional mobility, and insufficient institutional support (12). Sustaining and investing in this workforce is critical to advancing health across demographics, and investing in their professional development maximizes their impact on public health and strengthens the overall health system (13). Providing ongoing training, career pathways, and institutional support strengthens the public health workforce and CHW/Ps’ ability to continue educating and engaging their communities. High workforce retention rates can be achieved through meaningful compensation and professional growth opportunities.

To address the gap in research engagement, and to support CHW/Ps’ professional development, the Community Engagement Core at the Southern California Clinical and Translational Science Institute (SC CTSI) launched the *Fundamentals of Health Research* training program in January 2023. This innovative initiative equips CHW/Ps with foundational knowledge about clinical research and the skills to apply that knowledge by engaging their communities in meaningful discussions about research and the importance of participation in research. Rooted in community-engaged research principles, this program offers a scalable and adaptable model for building a more representative research workforce.

This paper describes the design and implementation of the *Fundamentals of Health Research* program, presents key findings from a mixed-methods evaluation, and identifies best practices for scaling and adapting this training using a community-engaged approach. The objectives of this evaluation were to:

1. Assess the knowledge retention of the CHW/Ps at the 3 month time point.
2. Understand changes in attitudes among the CHW/Ps about research participation and the research process following the training.
3. Understand the impact of the training on the CHW/Ps confidence in communicating about research to their communities.
4. Measure the real-world application of learned concepts through both education about research to their communities as well as rates of referrals to research studies.

## 2 Materials and methods

### 2.1 Program setting

The Southern California Clinical and Translational Science Institute (SC CTSI) is part of a national consortium of Clinical and Translation Science Award (CTSA) hubs funded through the National Institutes of Health (NIH), which are dedicated to accelerating the process of turning scientific discoveries into real-world treatments that improve health outcomes. SC CTSI supports researchers, clinicians, and community partners to enhance the impact and efficiency of translational research, and to ensure that research is accessible to populations who have historically had low rates of participation.

As part of SC CTSI, the Community Engagement (CE) Core serves as the bridge between academic researchers and local communities. We build sustainable partnerships, conduct consultations with researchers, and deliver community education and training programs that promote health and research literacy, counter misinformation, and build trust. A key strategy to increase research participation and ensure that communities are partners, not just participants in the research process is building community capacity. As such, this training program for CHW/Ps strengthens their understanding of the research process by equipping them with the knowledge and tools to educate their communities about research that aligns with and advances SC CTSI’s mission to expand research access, foster trust, and improve health outcomes.

### 2.2 Program development

The *Fundamentals of Health Research* training program was originally developed in 2016 by the Southern California Community Engagement Consortium, a collaboration of one healthcare organization and four academic institutions that are all CTA hubs. The objective of this consortium was to develop a program to build capacity among CHW/Ps to address the persistently low participation of minority populations in research. The curriculum was designed to train CHW/Ps to increase community knowledge about research, advocate for research, dispel myths and misinformation, and help community members understand and ultimately choose to participate in research. While the curriculum was designed by this consortium, it was further informed by and refined through focus groups with CHW/Ps with whom the participating academic institutions had worked. Additionally, it was the intention of the consortium that the curriculum and related activities could and should be modified as necessary to meet the needs of the CHW/Ps to which it was being delivered, which we did prior to launching this training in 2023.

Grounded in a community-engaged framework, this innovative program leverages the existing knowledge, skills, and trusted roles of CHW/Ps to increase community understanding of, and advocate for, research, encourage participation, and bridge the gap between researchers and communities with low rates of participation. The training empowers CHW/Ps to participate in recruitment and study design processes and to engage their communities in research discussions thereby serving as a bridge between communities and research institutions.

### 2.3 Program description

This 12-h training is delivered through a five-part curriculum that is structured sequentially to build foundational knowledge about health research. The training is delivered by experienced CHW/Ps from our Community Engagement team, who were also involved in the development of the training thus reinforcing their deep understanding and knowledge of the material. Additionally, they work in teams during the trainings, whereby one of the CHW/Ps is the facilitator, while the other is support staff. These roles alternate for each training, giving each a chance to facilitate and to observe the other, which helps to ensure standardization of the training across different sessions. The training, along with all related materials and surveys, is offered in English and Spanish and is adaptable to virtual, in-person, or hybrid formats depending on the needs of partner organizations and participants.

Each of the five lessons is aligned with specific learning objectives culminating in a final project in which participants design their own research project on a health topic of relevance to their community. Applying what they have learned, the participants complete their project by using a *Study Design Worksheet* template to develop a research question, determine the appropriate study design, define participant criteria, and outline recruitment strategies. The culmination of this process involves participants presenting their research projects in either a poster or PowerPoint format, with fellow trainees serving as a mock Institutional Review Board (IRB) to provide feedback and simulate real-world research review processes.

The curriculum is divided into five core lessons, each with specific learning objectives.

1. Introduction: Introduces the purpose and role of research in society. Participants learn key research terms, debunk common research myths, identify barriers to research participation and develop strategies for effective community engagement.
2. Types of Research: Explains how to distinguish between different research methods and understand the potential risks and benefits, highlighting how study design affects research outcomes and participant experiences.
3. What is Research: Builds on the previous lessons, to deepen the participant’s understanding of research and its significance. This lesson emphasizes addressing mistrust and misconceptions and how to overcome barriers to research participation by taking a culturally tailored approach.
4. Research Process: Provides an overview of how research studies are designed and conducted, including study timelines and the roles of various research team members.
5. Research Participant Protection: Focuses on research ethics, historical case studies, and current protections for research participants, such as regulations and oversight from the Institutional Review Board (IRB), National Institutes of Health (NIH), and The Food and Drug Administration (FDA).

#### 2.3.1 Innovative model

The training program employs an innovative, interactive model uniquely tailored for CHW/Ps. This program emphasizes a community-centered approach that is culturally relevant and sustainable, tailored specifically to meet the needs and leverage the unique position of CHW/Ps within their communities. A defining feature is its interactive methodology, which draws from evidence-based community training models, including the *Research Ambassador Training Program* (3), and is regularly updated to reflect evolving best practices and research standards.

The interactive components to the training include:

- Role-playing activities: Participants engage in structured role-play exercises to explore research concepts such as informed consent, IRB protocols, blinding, and risk–benefit analysis from the perspectives of both researchers and participants. These scenarios are followed by group discussions that reinforce understanding and encourage reflection.
- Study design homework: Participants complete a *Study Design Worksheet* as a homework assignment. This task requires them to develop a mock research study focused on a health issue relevant to their community. They outline their research question, choose a study design, define participant eligibility, and consider recruitment strategies and ethical concerns, potential risks and benefits. This activity builds critical thinking skills and research literacy by challenging participants to apply research design principles to real-world problems.
- Mock IRB presentation: On the final day of training, participants present their proposed research projects using a poster or PowerPoint presentation. Fellow trainees act as a mock Institutional Review Board (IRB), offering feedback in a simulated research review process. This component reinforces learning and introduces trainees to the ethics and logistics of research implementation.

### 2.4 Recruitment and participation

CHW/Ps were recruited through established partnerships with trusted community-based organizations that train or employ CHW/Ps. These community-based organization are embedded in the communities of South, Central, and the Eastside of Los Angeles, which are the communities in which our team works. The work of these organization ranges from social support for domestic violence survivors, housing advocacy, and healthcare. The active engagement of these organizations in local networks and community-based groups helped extend the program’s reach and visibility. Alumni of the program often served as ambassadors, sharing positive experiences with peers, promoting the training, and encouraging broader participation. This word-of-mouth recruitment strategy, grounded in trusted relationships, has been instrumental in expanding the program’s reach.

To participate, individuals must have prior experience as a CHW/P, either volunteer or paid, and be willing to commit to completing the full 12-h curriculum, related assignments, and presentation.

#### 2.4.1 Participant support and incentives

To foster an engaging and supportive learning environment, participants receive several resources and incentives. All trainees are provided with a binder of all training materials, which is mailed in advance to those attending virtual sessions. For in-person trainings, meals are provided to create a welcoming environment and promote comfort and community building.

Upon successful completion of the program, participants receive a certificate of completion and a gift card, both as recognition of their time, effort, and newly acquired skills. These incentives not only acknowledge participants’ dedication but also reinforce the value of their role in advancing community health and research literacy.

### 2.5 Evaluation

To evaluate the effectiveness of the *Fundamentals of Health Research* training program, we implemented a structured mixed-methods evaluation, integrating both quantitative and qualitative data to capture a comprehensive understanding of participant outcomes. Mixed-methods designs are well suited for evaluating community-based programs, offering both quantitative outcomes and qualitative insights into participant experiences (14, 15). This approach allowed us to assess knowledge acquisition, changes in attitudes, as well as confidence in communicating about research and the real-world application of learned concepts within communities. This project and all evaluation tools were approved by the Institutional Review Board at the University of Southern California. The evaluation framework included the following components:

- Pre-training survey: Administered prior to the start of the training. Collected baseline data on participants’ demographic characteristics, prior research experience, and foundational knowledge of clinical research, including research methods, participant rights, and common myths or misconceptions.
- Post-training survey: Administered immediately after the training. Assessed knowledge gains, changes in attitudes toward research, and participants’ self-reported confidence in discussing research with community members and their intent to use the information they had learned.
- Satisfaction Survey: Also completed immediately post-training. Evaluated participants’ satisfaction with the training format, content, and delivery.
- Three-month follow-up: Designed to measure short-term impacts including, knowledge retention, participants’ ability to apply the training content, and to identify any initial barriers or facilitators encountered when applying the training content in real-world settings.
- Twelve-month follow-up: Assessed the longer-term impact of the training, including success stories, continued engagement with research-related activities, and any ongoing barriers or facilitators to promoting research participation.

The primary outcomes of interest included: Change in knowledge of clinical research concepts; Change in attitudes and perceptions of research; Confidence and self-efficacy in discussing research with community members; Application of training content in real-world contexts.

Surveys were administered in English and Spanish, using digital formats for virtual sessions and paper formats for in-person sessions. For virtual sessions, participants received the pre-survey via email prior to the training, with reminders sent before and at the start of the session. Post-training and satisfaction surveys were also emailed, with up to three follow-up calls made to encourage completion. In-person participants completed pre- and post-surveys on paper during the training, with time allotted for completion and staff checking for completeness. All participants received follow-up surveys by email at 3 and 12 months post-training, with follow-up calls to boost response rates.

While the instruments were not formally pilot tested, face validity was established through expert review by survey methodology experts at SC CTSI and by the experienced CHW/Ps on our Community Engagement team. These individuals brought expertise in survey design and a deep understanding of the training content, delivery approach, and the communities with whom we work. This helped ensure the tools were clear, culturally and linguistically appropriate, and aligned with the experiences and needs of the CHW/Ps participating in the training. This process, also informed by previous community-based workshops on this topic (3), supported content validity by ensuring the measures reflected key dimensions of the training content and objectives.

By capturing both numerical trends and narrative feedback, this mixed-methods design provided a comprehensive understanding of how the training shaped participant learning, engagement, and action. The data also informed iterative improvements to the curriculum and guided future program planning.

#### 2.5.1 Data analysis

The process for data analysis differed for the quantitative and qualitative data. The quantitative analysis involved calculating descriptive statistics and average percent changes in participant knowledge scores at three time points: pre-training, immediate post-training, and 3-month follow-up. These scores provided a snapshot of knowledge acquisition and retention.

Qualitative responses from open-ended questions were reviewed and summarized using a rapid thematic analysis approach (16). These were captured at three time points: immediate post-training, 3-month follow-up, and 12-month follow-up, though our analysis was focused primarily on the 12-month follow up. Responses were then grouped by content similarity, and recurring themes were identified to capture participants’ experiences and perceptions of the training. Three members of the research team reviewed the qualitative data and then discussed to achieve consensus on the themes.

## 3 Results

In January 2023, the Community Engagement Core at SC CTSI launched the *Fundamentals of Health Research* training program. The first cohort began in March 2023, and by December 2023, 9 trainings had been conducted with a total of 196 CHW/Ps who completed the training. There was an average of 21 participants per training. This analysis focuses on the 128 CHW/Ps who completed their training between March and September 2023 (Table 1).

**Table 1:** Demographics.

	n	%
Gender		
Male	5	3.9%
Female	123	96.1%
Other	0	0.0%
Age		
18–25	3	2.3%
26–35	7	5.5%
36–45	37	28.9%
46–55	46	35.9%
56+	26	20.3%
No response	9	7.0%
Race/Ethnicity		
White	12	9.4%
Black/African American	2	1.6%
Latino/Hispanic	119	93.0%
Asian	0	0.0%
Pacific Islander/Native Hawaiian	0	0.0%
American Indian/Alaska Native	1	0.8%
Other	0	0.0%
Education		
Less than high school	44	34.4%
High school graduate/GED	33	25.8%
Some college	20	15.6%
Trade/Technical/Vocational school	16	12.5%
Bachelor’s degree	10	7.8%
Graduate school	5	3.9%
Employment status		
Employed-full-time	29	22.7%
Employed-part-time	49	38.3%
Unemployed–Student/Training/Stipend	20	15.6%
Unemployed	30	23.4%

Follow-up data were collected at 3 and 12 months post-training. Of the 128 participants, 103 (80.5%) completed the 12-month follow-up survey. Participants resided across 48 zip codes in Los Angeles County. Most identified as female (96%) and Latino/Hispanic (93%) and represented a wide range of educational and employment backgrounds, reflecting the varied experiences of CHW/Ps across the region.

### 3.1 Quantitative assessment

**Figure 1:**
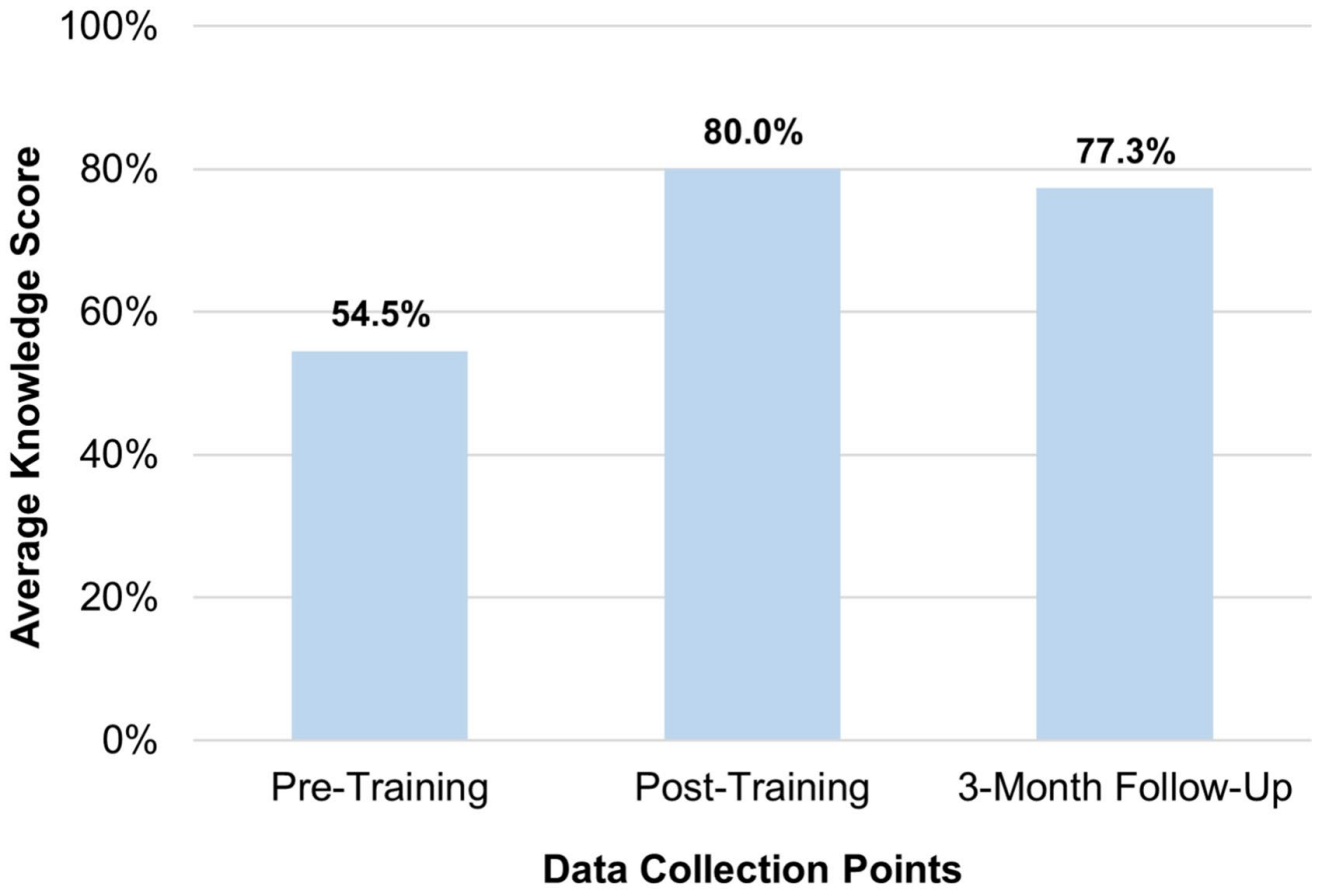
Average participant knowledge gain.

To assess the effectiveness of the training program, CHW/Ps responded to questions to assess their knowledge change, their confidence in utilizing what they learned, and the change in their intention to participate in research studies. From the pre-training to the post-training assessment, there was a notable 46.8% increase in average overall knowledge scores. At the 3-month follow up, only a slight percent decrease of −3.2% was observed, demonstrating retention of knowledge (Figure 1; Table 2).

**Table 2:** Knowledge gain: pre and post and 3-month follow up.

Knowledge question	Average pre-training score	Average post-training score	Percent change pre-post	Average 3-month follow-up score	Percent change post-follow-up
Clinical trials are used for:	62.5%	71.9%	15.0%	65.5%	−8.9%
What government agency approves drugs and devices?	78.1%	91.4%	17.0%	72.7%	−20.4%
There is significant representation of minority groups in clinical trials.	37.5%	60.9%	62.5%	58.2%	−4.5%
Medications and treatments can have different effects on people of different races/ethnicities.	72.7%	90.6%	24.7%	76.4%	−15.7%
The study investigator decides who receives the intervention (drug) and who receives the control (placebo).	44.5%	39.1%	−12.3%	58.2%	48.9%
For research to be ethical, all participants must choose to participate by going through an informed consent process.	77.3%	97.7%	26.3%	94.5%	−3.2%
Participation in research is optional, and a participant may choose to leave the study at any time.	71.9%	99.2%	38.0%	87.3%	−12.0%
Research participants must be treated with respect throughout the duration of their participation.	91.4%	100.0%	9.4%	96.4%	48.9%

#### 3.1.1 Twelve-month follow up

Participants rated their confidence in explaining key research concepts on a 10-point scale 1 (Not at all Confident) to 10 (Extremely Confident). The average overall confidence score was 8.0 out of 10.0 (Table 3), indicating a generally high level of confidence. Regarding intentions to participate in research, 49.1% of participants reported an increased likelihood of participation post-training, while only 5.5% reported a decrease.

**Table 3:** Twelve-month follow-up confidence scores (*n* = 103).

Confidence question	Average scores (1–10 scale)
How confident do you feel teaching community members about important health topics?	8.1
How confident do you feel explaining the potential benefits of participating in a clinical trial?	8.1
How confident do you feel explaining the potential risks of participating in a clinical trial?	7.7
Average Overall Confidence Score	8.0

At the 12-month follow-up, participants also reported how they have and continue to apply what they learned in both their personal and professional lives (Table 4). They were also asked about how they had applied what they had learned from the training to both their professional and personal lives including whether they had participated in any research studies since completing the training. In response to questions about how they had applied what they had learned, participants reported informing over 2,100 friends and family members, and more than 1,000 coworkers and colleagues about clinical research, underscoring the broad reach of CHW/Ps in their communities. Additionally, 54% reported referring people directly to research studies for which they could participate, thereby demonstrating their active involvement in promoting research participation.

**Table 4:** Twelve-month follow-up assessment of how training information was applied (*n* = 103).

How have you applied the information that you learned from the *Fundamentals of Health Research* training? (Select all that apply)	*n*	%
Conducted in-person community workshops	17	16.5%
Conducted virtual community workshops	7	6.8%
Shared the information with friends/family	95	92.2%
Shared the information with other *CHW/Ps* and co-workers	66	64.1%
Developed educational flyers/pamphlets/brochures to distribute	5	4.9%
Invited community members to workshops delivered by the Community Engagement program	36	35.0%
I do not know	1	1.0%
I have not	0	0.0%
Other (please specify below)	2	1.9%

Regarding the application of the training in their personal lives, almost 60% of participants had participated in, were participating in, or were enrolled in a research study (of these, 75% participated in a clinical trial). These results highlight the effectiveness and longer-term impact of this program.

### 3.2 Qualitative assessment

This section analyzes the qualitative data from the post-evaluation and the 12-month follow-up, which aimed to explore the outcomes, impacts, and potential improvements for the program. Key themes emerged from the participant feedback that illustrate the impact of this training.

#### 3.2.1 Key learnings

One of the most significant insights came from the question, “What was the most important thing you learned today?” Many participants highlighted an increased understanding of their rights as research subjects, particularly regarding informed consent and privacy protections. This newfound knowledge helped reduce fears and built confidence to participate in research. One participant shared their experience of personally participating in studies and actively encouraging others to join, stating:

“*I have been able to participate personally, and I have brought 6 family members to participate in a study that analyzed hereditary diseases in Latinos. I also shared the flyer with more than 20 people to encourage them to participate […] Our teenage son participated in a study, and I shared the information with more than 40 mothers to encourage them to have their children participate […] I have only motivated the community to participate in these studies, and I know that many of the people with whom I have shared the information have participated.*”

In addition to these insights, participants emphasized the need for greater Hispanic/Latino representation in research as a crucial step in addressing health disparities. The training also significantly improved participants’ comprehension of the research process, particularly in terms of study structure and the ethical considerations inherent in research.

#### 3.2.2 Community impact

Participants also recognized the broader societal impact of research, including the potential to improve healthcare outcomes and advance medical science. This understanding drove their desire to contribute to research efforts aimed at improving health and addressing community needs. The training also helped dismantle prevalent myths surrounding research participation, particularly in terms of clinical trial safety and integrity.

The 12-month follow-up provided further evidence of the training’s long-term impact. When asked about the broader effects of the training, participants shared success stories where their community members had made informed health decisions and adopted healthier behaviors due to their increased understanding of research. CHW/Ps used their training to foster greater trust in their communities by addressing fears and debunking misinformation about research participation. These efforts included organizing community health events and focus groups to promote discussions about health research and raise awareness. As one participant stated, “I gained the trust of a group from the Church of Jesus Christ of Latter-day Saints to present a topic at their monthly meetings with 30 to 35 people. I have already been there three times. I have two more community groups awaiting dates for me to give presentations… Personally, it has been a success for me to teach…where I also put into practice what I have learned with you.”

#### 3.2.3 Identifying and addressing barriers

Some barriers remained in the promotion of clinical trials. Participants reported challenges related to mistrust, stemming from historical concerns about medical institutions and exploitation. Misinformation, cultural and language differences, and logistical issues such as time constraints and competing life priorities also emerged as significant obstacles. One participant who has been educating her community about research expressed that:

“*A large part of the problem with the lack of community participation in these types of clinical trials is the lack of knowledge about the procedure and the purpose of the trials. By educating the population, we can change people’s way of thinking because, as one woman told me in one of the trainings: ‘I always thought it was just for using them, but now I understand that much of the medical progress is thanks to these types of studies.’ The ability to hear that at least one person’s perception has changed and that they will now share the information with their family is a great step forward*.”

Additionally, concerns about immigration status and reluctance to embrace new health research methods highlighted the need for targeted strategies to overcome these barriers and further engage the community in research participation.

These findings emphasize the importance of continuous evaluation and adaptation of the training programs to meet community needs, address barriers to participation, and strengthen the capacity of CHW/Ps to support community engagement in health research.

## 4 Discussion

The *Fundamentals of Health Research* training program leverages the unique strengths of CHW/Ps to enhance community engagement in public health research. This innovative initiative aimed to strengthen the capacity of CHW/Ps in engaging community members around clinical research, as well as enhance health and research literacy, encourage participation in clinical trials, and address underrepresentation in medical research. By equipping CHW/Ps with the knowledge and tools to understand and effectively communicate research concepts, the program enabled them to simplify complex information, foster trust, and advocate for community involvement in research efforts.

CHW/Ps are trusted, deeply embedded members of their communities (5, 13). They are frequently approached with questions about a range of health issues, including research, and are uniquely positioned to serve as cultural and linguistic bridges (13, 17). This training builds on their existing skillsets and relationships by providing advanced, research-specific knowledge. As a result, CHW/Ps are better equipped to respond to community concerns, dispel myths and misinformation, and support individuals in understanding and participating in research studies. In many cases, they even act as informal patient navigators, guiding others through the research process.

A key outcome of this initiative was the increased confidence and capacity of CHW/Ps to educate and facilitate meaningful engagement around research. The training not only enhanced CHW/Ps knowledge but also strengthened the link between community priorities and research opportunities, creating a more supportive and informed environment for participation.

Ultimately, the program served as a vital platform for education, empowerment, and advocacy. By investing in CHW/Ps as community educators and research ambassadors, this model holds significant promise for advancing health across demographic populations, building research literacy, and ensuring that all voices are included in shaping the future of health research.

### 4.1 Best practices

Several best practices emerged during the implementation of the *Fundamentals of Health Research* program that reinforce, and further support key components of community engaged research. Building trust is essential. Begin by collaborating with trusted community organizations. Delivering the training in partnership with five community-based organizations, including one that incorporated the curriculum into its ongoing programs, significantly increased credibility, participation, and long-term sustainability. Second, invest in long-term relationships. Ongoing communication with CHW/Ps and organizational partners allowed the training to remain responsive and relevant to evolving community needs. Third, ensure that all material is culturally tailored and language specific. Training and research teams that reflect the identities, languages, and lived experiences of the communities they serve can play a critical role in fostering trust and engagement (7). Fourth, provide compensation for their participation. This recognizes their time and expertise and strengthens mutual respect. Finally, incorporate community feedback. Evaluations enabled the curriculum to adapt, thereby increasing its cultural relevance and effectiveness.

These best practices align with broader principles of community-engaged research, which emphasize trust, mutual benefit, and shared ownership. They also highlight the importance of positioning community members not just as participants, but as co-creators and collaborators in public health solutions.

### 4.2 Limitations

The program has demonstrated early success, however certain limitations provide valuable insight for future development. First, the evaluation relies on self-reported data from participants, which may be subject to bias. While self-reporting remains a standard and practical method for assessing short-term outcomes such as knowledge acquisition and changes in attitudes, it may not translate into actual medium-term or long-term outcomes, or community impact. To address this, we collected data at multiple time points, including 12-month follow-ups to provide a more nuanced understanding of outcomes over time. Additionally, while we achieved an 80% response rate by 12-months, we acknowledge that attrition could impact the findings, and therefore generalizability. Future iterations could incorporate additional objective measures, such as structured interviews or case studies, to validate and enrich the findings.

Second, the training has thus far been delivered exclusively in Spanish to CHW/Ps narrowing its immediate applicability to non-Spanish speaking communities, thereby also limiting its generalizability. However, given the historically low research participation rates among Spanish-speaking communities, this focus represents a strategic starting point. There is considerable potential to adapt and expand the curriculum to other demographic groups through community-engaged, co-development processes that ensure it is culturally tailored and language appropriate (1). Such adaptations could include historical and contemporary examples specific to the target community’s experiences with healthcare and research.

Finally, our ultimate hope is that this training can be scaled and can create formal professional pathways for CHW/Ps to maximize long-term impact.

### 4.3 Public health implications

The *Fundamentals of Health Research* training program meaningfully contributes to the public health field by expanding the capacity of CHW/Ps to serve as research educators and advocates. CHW/Ps trained through this program are uniquely positioned as trusted members of their communities to educate, dispel myths and misinformation, and promote participation in research. The CHW/Ps can also be essential contributors to healthcare teams and research staff. By creating additional professional pathways for CHW/Ps the program has the potential to impact the overall health and wellbeing of community members, exemplifying the core values of public health advancement. Continued support and development of such trainings are essential for reducing health disparities and improving access to public health interventions.

### 4.4 Conclusion

The *Fundamentals of Health Research* training program offers an innovative, scalable community engaged model for bridging the gap between research institutions and communities. Rooted in a community-engaged approach, this model promotes sustainable partnerships, builds trust, and supports long-term investment in building capacity in the CHW/Ps workforce. It is adaptable to a range of community contexts through co-design, linguistic and cultural tailoring, and mixed-methods evaluation. Continued support for community-driven, evidence-based training initiatives is critical to improving research access, participation, and outcomes for all communities.

## Data Availability

The raw data supporting the conclusions of this article will be made available by the authors, without undue reservation.
